# Providers’ Perceptions and Training Needs for Counseling Adolescents Undergoing Voluntary Medical Male Circumcision

**DOI:** 10.1093/cid/cix1036

**Published:** 2018-04-03

**Authors:** Aaron A R Tobian, Kim H Dam, Lynn M Van Lith, Karin Hatzold, Arik V Marcell, Webster Mavhu, Catherine Kahabuka, Lusanda Mahlasela, Eshan U Patel, Emmanuel Njeuhmeli, Kim Seifert Ahanda, Getrude Ncube, Gissenge Lija, Collen Bonnecwe, Michelle R Kaufman

**Affiliations:** 1 Department of Pathology, Johns Hopkins University School of Medicine, Baltimore, Maryland; 2 Johns Hopkins Bloomberg School of Public Health, Baltimore, Maryland; 3 Johns Hopkins Center for Communication Programs, Baltimore, Maryland; 4 Population Services International, Harare, Zimbabwe; 5 Department of Pediatrics, Johns Hopkins University School of Medicine, Baltimore, Maryland; 6 Centre for Sexual Health & HIV/AIDS Research, Harare, Zimbabwe; 7 CSK Research Solutions, Ltd., Dar es Salaam, Tanzania; 8 Centre for Communication Impact, Pretoria, South Africa; 9 Office of HIV/AIDS, Global Health Bureau, United States Agency for International Development, Washington, District of Columbia, Washington D.C; 10 Ministry of Health and Child Care, Harare, Zimbabwe; 11 Ministry of Health, Community Development, Gender, Elderly and Children, Dar es Salaam, Tanzania; 12 National Department of Health, Pretoria, South Africa

**Keywords:** voluntary medical male circumcision, sub-Saharan Africa, adolescent boys, counseling, HIV prevention

## Abstract

**Background:**

The majority of individuals who seek voluntary medical male circumcision (VMMC) services in sub-Saharan Africa are adolescents (ages 10–19 years). However, adolescents who obtain VMMC services report receiving little information on human immunodeficiency virus (HIV) prevention and care. In this study, we assessed the perceptions of VMMC facility managers and providers about current training content and their perspectives on age-appropriate adolescent counseling.

**Methods:**

Semistructured in-depth interviews were conducted with 33 VMMC providers in Tanzania (n = 12), South Africa (n = 9), and Zimbabwe (n = 12) and with 4 key informant facility managers in each country (total 12). Two coders independently coded the data thematically using a 2-step process and Atlas.ti qualitative coding software.

**Results:**

Providers and facility managers discussed limitations with current VMMC training, noting the need for adolescent-specific guidelines and counseling skills. Providers expressed hesitation in communicating complete sexual health information—including HIV testing, HIV prevention, proper condom usage, the importance of knowing a partner’s HIV status, and abstinence from sex or masturbation during wound healing—with younger males (aged <15 years) and/or those assumed to be sexually inexperienced. Many providers revealed that they did not assess adolescent clients’ sexual experience and deemed sexual topics to be irrelevant or inappropriate. Providers preferred counseling younger adolescents with their parents or guardians present, typically focusing primarily on wound care and procedural information.

**Conclusions:**

Lack of training for working with adolescents influences the type of information communicated. Preconceptions hinder counseling that supports comprehensive HIV preventive behaviors and complete wound care information, particularly for younger adolescents.

Voluntary medical male circumcision (VMMC) reduces the risk of human immunodeficiency virus (HIV) and other sexually transmitted infections (STIs) [1–13]. In 2011, the World Health Organization (WHO) and UNAIDS launched the joint strategic action framework for acceleration of the scale-up of VMMC, aimed at reaching 80% of men aged 15–49 years in sub-Saharan Africa to curb the HIV epidemic [14–16]. Younger adolescent clients (10–14 years) are also receiving VMMC in the priority countries [17]. VMMC provides a unique forum to educate young males not only about a range of sexual and reproductive health issues, including HIV preventive behaviors and gender issues, but also to link them into care and treatment, if necessary [18, 19]. The WHO minimum package recommends providers, regardless of client age, deliver HIV testing services, HIV risk reduction strategies, information on VMMC risks and benefits, and instructions on wound care [18, 20].

Despite these recommendations, it is unclear if adolescents (10–19 years), especially younger males (<15 years), are receiving the same range of counseling regarding the procedure, wound care, and/or other sexual and reproductive health education as their adult counterparts [21]. In 3 priority countries, HIV and sexual health counseling and in-service communication were found to be largely absent during VMMC services for adolescents, particularly those aged <15 years [19, 22]. This may be due to lack of specific guidance and providers’ belief that sexual health information and risk reduction discussions were unnecessary for younger clients who they assumed had not reached sexual debut.

Little is known about the type of training providers receive and their approach to applying their training and knowledge. The purpose of the current study was to explore provider perceptions of past VMMC counselor training and reported strategies for counseling adolescents as compared to adult clients, and facility managers’ views of their support to providers working with adolescents in 3 countries.

## METHODS

The Tanzania National Institute for Medical Research, the Human Sciences Research Council in South Africa, the Medical Research Council of Zimbabwe, and the Johns Hopkins Bloomberg School of Public Health Institutional Review Board approved the study prior to data collection.

### Setting and Participants

In-depth, semistructured interviews, tailored for each group, were conducted with providers (eg, VMMC counselors, nurses, midwives) who deliver information to adolescent males seeking VMMC services and with facility managers who oversee the provision of VMMC services to adolescent males. Data were collected in South Africa (February 2016–June 2016), Tanzania (June 2015–September 2015), and Zimbabwe (August 2015–December 2015). Research field supervisors visited facilities that offer VMMC services to adolescents to inform VMMC facility managers and staff about the research and to request participation. Some sites were permanent health facilities, such as hospitals and community clinics, while others were mobile clinics, such as medical tents temporarily constructed in order to offer services in a given community before moving on to other settings [23]. The locations of the 12 service sites were rural (4), peri-urban (3), and urban (5). The study recruited female and male VMMC providers who primarily counseled adolescent males aged 10–19 years and facility managers who oversaw VMMC service provision for adolescent males at the selected facilities.

### Procedures

Individual informed consent was obtained before interviewing eligible providers and facility managers. Local research field workers conducted interviews in the countries’ local languages (Sesotho, isiZulu, or isiSwati in South Africa; kiSwahili in Tanzania; Shona or Ndebele in Zimbabwe) or in English if the participant preferred. All interviews were audio recorded, transcribed, and translated into English for coding and analysis.

Interviews with providers focused on the VMMC counseling process, the providers’ knowledge and training specific to VMMC, and strategies for counseling adolescent and adult clients. Interviews with facility managers explored the application of existing VMMC guidelines, as well as availability and description of provider training, resources, and other structural factors that may influence the quality of VMMC service delivery for adolescents.

### Analyses

Two coders independently coded the data using a 2-step process and Atlas.ti qualitative coding software (Berlin, Germany), as previously described [19]. First, the 2 coders read through all transcripts independently and identified organizational categories. Discrepancies between their identification of categories were discussed until a consensus was met. The coders then applied the final list of categories to all transcripts and identified themes under each category to help further organize the data. The coders compared their interpretations and discussed individual coded text for all manuscripts before reaching a consensus. In the rare event that coders could not come to a consensus, the Principal Investigator made the final decision. We present the findings following the organizational categories identified in the first step of the analysis and describe the substantive themes within each category.

## RESULTS

### Participant Characteristics

Demographic information for both providers and facility managers is summarized in Table 1. A total of 33 (South Africa = 9, Tanzania = 12, Zimbabwe = 12) interviews were conducted with VMMC providers. The providers' mean age was 41.0 years, and 78.8% were female. Providers consisted of nurses/midwives (60.6%), counselors (33.3%), and other healthcare workers (6.1%). Overall, they had an average of 3.9 years of adolescent VMMC service experience. In addition to counseling, providers reported that it was their responsibility to deliver the following services: HIV testing (84.8%); family planning, including provision of condoms (48.5%); STI testing and treatment (42.4%); and other general health services (45.5%).

**Table 1.** Study Participant Demographics by Country

Characteristics	Total, N (%) or M (SD)	South Africa, n (%) or M (SD)	Tanzania, n (%) or M (SD)	Zimbabwe, n (%) or M (SD)
**Provider**	N = 33	N = 9	N = 12	N = 12
Gender				
Female	26 (78.8)	7 (77.8)	12 (100)	7 (58.3)
Male	7 (21.2)	2 (22.2)	0 (0.0)	5 (41.7)
Age, y (mean, SD)	41.0 (9.9)	35.4 (9.1)	46.2 (9.3)	39.9 (9.0)
Provider type				
Nurse/midwife	20 (60.6)	1 (11.1)	8 (66.7)	11 (91.7)
Counselor	11 (33.3)	7 (77.8)	3 (25.0)	1 (8.3)
Other (field recruiter, clinical officer)	2 (6.1)	1 (11.1)	1 (8.3)	0 (0.0)
Facility type				
Public	21 (63.6)	3 (33.3)	9 (75.0)	9 (75.0)
Other (nongovernmental, mixed, faith-based)	12 (36.3)	6 (66.7)	3 (25.0)	3 (25.0)
Facility area				
Urban	16 (48.5)	3 (33.3)	4 (33.3)	9 (75.0)
Peri-urban	7 (21.2)	2 (22.2)	5 (41.7)	0 (0.0)
Rural	10 (30.3)	4 (44.4)	3 (25.0)	3 (25.0)
Other provider responsibilities^a^				
Only VMMC	4 (12.1)	2 (22.2)	0 (0.0)	2 (16.7)
General health services	15 (45.5)	3 (33.3)	9 (75.0)	3 (25.0)
HIV testing	28 (84.8)	7 (77.8)	12 (100)	9 (75.0)
HIV treatment or care	7 (21.2)	1 (11.1)	4 (33.3)	2 (16.7)
STI testing and treatment	14 (42.4)	4 (44.4)	4 (33.3)	6 (50.0)
Family planning^b^	16 (48.5)	3 (33.3)	7 (58.3)	6 (50.0)
Child health	8 (24.2)	0 (0.0)	5 (50.0)	3 (25.0)
Years of adolescent VMMC experience (mean, SD)	3.9 (2.0)	4.6 (3.4)	3.9 (0.8)	3.4 (1.5)
**Facility manager**	N = 12	N = 4	N = 4	N = 4
Gender				
Female	5 (41.7)	3 (75.0)	1 (25.0)	1 (25.0)
Male	7 (58.3)	1 (25.0)	3 (75.0)	3 (75.0)
Age of facility manager, y (mean, SD)	42.4 (11.7)	43.3 (17.2)	40.3 (4.0)	43.8 (13.3)
Manager type				
Doctor	2 (16.7)	0 (0.0)	1 (25.0)	1 (25.0)
Nurse	5 (41.7)	2 (50.0)	2 (50.0)	1 (25.0)
Other (director, etc.)	5 (41.7)	2 (50.0)	1 (25.0)	2 (50.0)
Years of experience as facility manager (mean)	6.3 (6.2)	2.1 (1.4)	8.8 (3.4)	8.1 (9.5)

Abbreviations: HIV, human immunodeficiency virus; M, mean; SD, standard deviation; STI, sexually transmitted infection; VMMC, voluntary medical male circumcision.

^a^Providers could provide multiple responses regarding their responsibilities.

^b^Including the provision of condoms.

Five of 12 facility managers were female. Facility managers had a mean age of 42.4 years. A majority of facility managers (83.3%) were head nurses or held director positions. Overall, they had an average of 6.3 years of facility management experience. Half of the facility managers also reported having direct responsibilities related to VMMC counseling, conducting or assisting with VMMC procedures, or HIV testing.

### Counseling Approach for Adolescents

When asked how counseling was approached in daily practice with adolescent clients compared to adult clients, providers and facility managers in all 3 countries articulated that the differentiation was not necessarily adolescent vs adult but rather young nonsexually active adolescent compared to older adolescent/adult (Table 2). Many providers felt it was important to hold back some details perceived to be irrelevant (eg, sexual health and HIV) for clients aged <15 years. These topics could be broached with older adolescents if the provider deemed it appropriate. Facility managers in all 3 countries indicated that their facilities generally conducted group counseling sessions according to age and engagement in sexual activity, often grouping younger adolescents (aged <15 years) separately from those aged >15 years.

**Table 2.** Identified Themes From Providers’ and Facility Managers’ Perspectives on Working With Adolescent Clients

Category	Theme	Specific Issues
Counseling approach for adolescents	Counseling younger adolescents (aged <15 years)	• Assumed not sexually active • Let adolescents drive information shared • Post-procedure care information often too advanced
	Counseling older adolescents	• Lack of consistency about what age to start sexual and reproductive health education (eg, ages 16+ years or 18+ years ) • Older males had more questions in general about sex, so providers felt it more appropriate to address sexual topics
Provider training	Lack of adolescent-specific training	• Training not consistently provided • Trainings often too general and for all clients • Limited training content
	Limited training capacity or refresher courses	• Variation in scope of training receipt • Lack of training within different age groups of adolescents • Team meetings occasionally used to identify gaps in training
Recommendations for provider training on counseling the younger male		• Emphasized need for improvements in the area of counseling the younger male • Addressed the need for adolescent-specific guidelines (on condom use, sexually transmitted infection care and treatment, and HIV counseling in general and specific to disclosing HIV-positive test results to younger clients)

Abbreviation: HIV, human immunodeficiency virus.

#### Counseling Younger Adolescents (<15 Years Old)

Providers and facility managers largely believed that very young boys (10–12 years) “don’t know much yet” and have fewer sexual experiences, so the counseling does not have to address sexual issues in detail or at all.

…the information that we talk about especially with this younger group [10-year-olds] is not much about people who have...these people they have not yet indulged [in sex] so we will mainly be focusing on the wound care, on hygiene, not much on like somebody who has...who has indulged. [Facility Manager, Zimbabwe]

Often, providers talked about approaching young adolescents by asking them what they already know about VMMC and letting their current knowledge and misconceptions drive the counseling process. While some providers did acknowledge that national guidelines, such as those in Tanzania, require them to address all content related to wound care, HIV prevention, and sexual health with all age groups, in practice providers appear to make decisions on content based on the age and assumed sexual experience of clients.

The young ones do not even know what condoms are; although the guidelines tell us to discuss condoms even with children, we do not discuss them with young children. [Provider, Tanzania]

Both providers and facility managers viewed post-procedure care information as being too advanced for younger boys, preferring to share this information with parents or guardians when available.

#### Counseling Older Adolescents

Providers and facility managers generally felt it was more appropriate to address sexual topics with older adolescents (>15 years) because they were more likely to have started experimenting with their sexuality, although a few providers thought sexual content was only appropriate for those aged ≥18 years.

We tell the older ones [18-19 years] to keep being faithful and abstain from sex. If they fail to do this then they should have one sex partner and always use condoms….I tell those who are 10-15 years not to have sex. For those who are 16 years or older, it is a bit tricky. At that age, many of them are going through puberty, and they tend to try out sex. Therefore, we tell them to do their best to abstain from sex, but if they fail, then they should always use condoms. [Provider, Tanzania]

Compared to counseling younger adolescents, providers felt they were more equipped to discuss a broader range of sexual topics with older boys, in part because older males had more questions related to sex post-VMMC, while younger boys’ questions focused on pain and details of the procedure.

### Provider Training

Providers and facility managers discussed the need for refresher trainings to keep abreast of accurate and comprehensive information regarding HIV and VMMC. They emphasized incorporation of training on age-appropriate HIV health education and counseling approaches, including communicating HIV-positive test results to adolescents.

#### Lack of Adolescent-Specific Training

Providers and facility managers reported that training on adolescent VMMC counseling and adolescent sexual and reproductive health was not consistently provided. In lieu of such training, providers said that they drew from past experiences and other trainings when providing adolescent VMMC counseling.

I was trained in VCT [HIV voluntary counseling and testing] before the PITC [provider-initiative testing and counseling] training. There are some techniques that I got from the VCT training, some things that are not even in the VMMC guidelines, but I do them anyways because the situation requires me to. We are allowed to add a few other things as long as we do not leave any gaps in the VMMC guidelines that we are supposed to follow; the major aim is to serve the client in the best way we can. [Provider, Tanzania]

In South Africa and Tanzania, providers stated that the trainings were too general for all clients. They were not instructed on how to counsel adolescents any differently from adult clients, other than to focus on building rapport with adolescents to gain their trust; to speak in a way easily understood by younger clients; and to make sure adolescents were accompanied by parents.

For the young ones we were trained on how to approach them at the very beginning [of the VMMC process]. They taught us to improve the way we counsel and deliver the message so that young males understand well. [Provider, Tanzania]

Similarly, facility managers in all 3 countries reported that the VMMC training curriculum for providers was generalized and not age specific and had limited focus on adolescents.

No, no we are not trained [on how to talk about sexual reproductive health issues, like sexual debut, STIs, condom use, sexual violence] those are the things that I can say maybe from the training that we had from tertiary education, yes we have that knowledge but in terms of specific training that is put in place to say go and do this training for adolescents there is nothing or refresher course or anything, no. [Facility manager, South Africa].

Facility managers noted that the training content pertaining to adolescents was limited mostly to VMMC age requirements, the consenting protocol, and the proper VMMC procedure for adolescents. However, compared to accounts from providers in other countries, providers and facility managers in South Africa did mention that the training included specific HIV counseling for adolescents. However, those who had received past training that included adolescent-specific approaches judged it insufficient in scope.

#### Limited Training or Refresher Courses

Facility managers in all 3 countries indicated that providers receive training on the full VMMC service package from a variety of sources, both governmental (department or Ministry of Health) and nongovernmental. However, providers generally reported being trained just once on VMMC counseling (sometimes 4 to 6 years ago). A small number of providers said they had occasionally received an update in training to then share with colleagues. In Zimbabwe, some providers said they were trained only once before offering VMMC services. The counseling content addressed how to counsel adolescents regarding VMMC and HIV; however, several respondents reported that they would feel more confident and comfortable when working with adolescents if they had more thorough and in-depth training.

Mainly the trainings we were doing were around adults. For the adolescents… really, we weren’t doing much. But the one [training] I got for the adolescent sexual and reproductive health…Yes, they will be giving us information, but it will be so brief that you don’t know how really to do it. [Provider, Zimbabwe]

Facility managers discussed this deficit of in-depth provider training and how adolescent training content has not been available to all staff.

There is a certain training which was conducted by the Ministry of Health about how to communicate with adolescents. I do not remember well, there were some people here who attended, it could be better if they could bring that training to us all. [Facility Manager, Tanzania]

In South Africa and Tanzania, facility managers mentioned working together with their staff to identify gaps in their training and using staff meetings or facility-based trainings as in-service programs.

### Recommendations for Provider Training on Counseling the Younger Male

Providers and certain facility managers emphasized the need for improvements in the counseling of younger males and provided examples on how to do so. For example, providers discussed needing adolescent-focused guidance in their VMMC training on specific content related to condom use, STI care and treatment, and HIV counseling, in general, and specific to disclosing HIV-positive test results to younger clients. They felt this would make them more comfortable speaking with and counseling younger clients.

We teach the children about HIV, but I think this is a bit higher than them…there must be a language that we can use with children and a language which we can use with adults. The language used in the guidelines is sufficient for adults, but I stammer when I talk to children. [Provider, Tanzania]

I think to train the service providers on child counseling would really help. Just getting the skills on how to deal with these adolescents and also on HIV issues, because the other reasons why we are not disclosing [their HIV status] maybe could be because we are not trained. When they come with their guardians, we sometimes refer them to family support because we feel they are better trained in child counseling. [Provider, Zimbabwe]

The need for adolescent-specific training was reinforced by facility managers.

People [providers] also need to be trained in adolescent sexual and reproductive health so that people [providers] are well-versed with things that affect young people. [Facility Manager, Zimbabwe]

Facility managers and providers agreed that VMMC training needs to incorporate thorough adolescent-specific recommendations to be effective.

## DISCUSSION

Overall, VMMC providers and facility managers interviewed in 3 countries reported having received little, if any, training on how to work with adolescent clients, especially younger clients. It is important for VMMC staff to be trained on how to properly assess the client’s sexual activity level, to frame counseling around the individual client’s needs, and to ensure that counseling is comprehensive for each adolescent client, regardless of their age or sexual experience.

The study findings in all 3 countries, which revealed training deficits on the needs of adolescents can have a potential negative impact on care, is supported by previous studies. A small qualitative study in South Africa suggested that clinics were not prepared to handle youth HIV counseling and testing [24]. A systematic review of general HIV interventions found that counseling for older adolescents may not be relevant or effective for younger adolescents [25]. A recent systematic review of best practices in adolescent VMMC found a general absence of health services addressing the specific needs of male adolescents [21]. The review suggested that barriers included incomplete information provision to adolescents, infrastructure limitations, stigmatization of sexuality, patient privacy violations, and fear of pain associated with the procedure, all of which can make the VMMC experience less than desirable for adolescents. The same review showed that factors linked to an effective experience for adolescents included engagement with parents and the community, an adolescent-friendly service environment, and use of age-appropriate VMMC materials that can be easily understood by young males.

This study has some limitations. Qualitative data, by nature, are not generalizable beyond the included participants. The study did not account for cultural differences among countries or between sites within countries but rather focused on the training and the counseling prescribed by WHO or local governments for adolescent VMMC clients. VMMC sites were identified in conjunction with the Ministry of Health in each country and the organizations conducting the procedures. The counseling approaches used and the experiences of adolescents might differ between sites, geographic locations, and the organizations managing the sites and providers. In addition, VMMC guidelines for counseling vary between countries.

For many young males, VMMC is their first contact with the health delivery system. The current lack of adolescent-specific guidelines and appropriate training curricula creates a challenge to adequate counseling by VMMC providers and facility managers for adolescents of varying ages about HIV preventive behaviors and risk reduction strategies. This situation leaves VMMC service providers to rely on their own best judgment to determine what approach is deemed appropriate. In the absence of clarity, each provider may be assessing clients using a different set of criteria, leaving little to no consistency within the VMMC counseling experience. VMMC programs may also consider having certain providers who are more comfortable communicating with younger adolescents about their sexuality dedicated to working with this clientele. Adolescent VMMC counseling is a neglected gateway to engage young clients in ongoing sexual and reproductive healthcare and falls short of delivering the WHO minimum package of services. If VMMC services are to be responsive to adolescent clients and prepare them for a lifetime of HIV-preventive and health-seeking behaviors, additional training related to both adolescent-specific messaging and age-specific counseling skills is essential.

## Notes

***Acknowledgments.*** We are grateful to the adolescent participants in this study for sharing their perspectives and experiences. The authors also thank the VMMC facility managers, community mobilizers, and providers for their support. Further, the authors acknowledge the contributions of the Technical Advisory Group for the adolescent VMMC assessment and their guidance throughout the study. We are privileged to work with this group of dedicated professionals from the US President’s Emergency Plan for AIDS Relief (PEPFAR), Centers for Disease Control and Prevention, US Department of Defense, the World Health Organization, United Nations Children’s Fund, and the Bill and Melinda Gates Foundation. Thank you to the Human Sciences Research Council in South Africa, CSK Research Solutions in Tanzania, PSI/Zimbabwe, and the Centre for Sexual Health & HIV/AIDS Research in Zimbabwe for their assistance with data collection. The authors also appreciate the assistance of Meaghen Murphy with copy editing and Maria Elena Figueroa with project start-up.

***Financial support.*** This work was supported by the United States Agency for International Development with PEPFAR funding (cooperative agreement AID-OAA-A-12-00058) to the Johns Hopkins Center for Communication Programs and cofunded by the UK Department of International Development through the Integrated Support Program in Zimbabwe.

***Supplement sponsorship.*** This article appears as part of the supplement “Adolescent Voluntary Medical Male Circumcision: Vital Intervention Yet Improvements Needed,” sponsored by Johns Hopkins University.

***Potential conflicts of interest.*** All authors: No reported conflicts of interest. All authors have submitted the ICMJE Form for Disclosure of Potential Conflicts of Interest. Conflicts that the editors consider relevant to the content of the manuscript have been disclosed.
